# Women’s voices on social media: the advent of feminist epidemiology?

**DOI:** 10.1186/s12982-021-00097-1

**Published:** 2021-06-16

**Authors:** Céline Miani, Yudit Namer

**Affiliations:** School of Public Health, Bielefeld University, Universitätstraße 25, 33615 Bielefeld, Germany

**Keywords:** Social media, Feminist epidemiology, Intersectionality, Inclusion

## Abstract

**Background:**

Social media have in recent years challenged the way in which research questions are formulated in epidemiology and medicine, and in particular when it comes to women’s health. They have contributed to the emergence of ‘new’ public health topics (e.g. gynaecological and obstetric violence, long-Covid), the unearthing of testimonials of medical injustice, and in some cases, the creation of new evidence and changes in medical practice.

**Main text:**

From a theoretical and methodological perspective, we observe two powerful mechanisms at play on social media, which can facilitate the implementation of feminist epidemiological research and address so-called anti-feminist bias: social media as a ‘third’ space and the power of groups. Social media posts can be seen as inhabiting a third space, akin to what is said off the record or in-between doors, at the end of a therapy session. Researchers somehow miss the opportunity to use the third spaces that people occupy. Similarly, another existing space that researchers are seldom interested in are peer-groups. Peer-groups are the ideal terrain to generate bottom-up research priorities. To some extent, their on-line versions provide a safe and emancipatory space, accessible, transnational, and inclusive. We would argue that this could bring feminist epidemiology to scale.

**Conclusion:**

Given the emancipatory power of social media, we propose recommendations and practical implications for leveraging the potential of online-sourced feminist epidemiology at different stages of the research process (from design to dissemination), and for increasing synergies between researchers and the community. We emphasise that attention should be paid to patriarchal sociocultural contexts and power dynamics, the mitigation of risks for political recuperation and stigmatisation, and the co-production of respectful discourse on studied populations.

## Social media and epidemiological research

Social media have in recent years challenged the way in which research questions are formulated in epidemiology and medicine, and in particular when it comes to women’s health. By giving a platform to those whose bodies and experiences have been long ignored or considered less important, and for whom conventional meeting spaces are not accessible, social media have contributed to the emergence of ‘new’ public health topics, the unearthing of testimonials of medical injustice, and in some cases, the creation of new evidence and changes in medical practice. The use of social media has been emancipatory, and new perspectives and resultantly new evidence have all emerged out of emancipatory work and paradigms.

## Online collective action

This can be seen through high profile examples of collective action: in the UK, Facebook groups acted as a catalyst for testimonies on the painful side effects of vaginal mesh supposed to treat urinary incontinence, which in turn led to class actions and new guideline for the use of such devices. In France, the very popular Twitter #payetonuterus and #payetongyneco on gynaecological and obstetric violence resulted in the production of a governmental report and the creation of an observatory to tackle this phenomenon [1]. One could see in these examples a fertile ground for the long-overdue advent and scaling up of what Inhorn and Whittle characterised twenty years ago as ‘feminist epidemiology’ [2]. As an alternative paradigm for social epidemiology, feminist epidemiology aims to tackle anti-feminist bias in research and deconstruct social hierarchies based on gender, racialisation, and class.

Unsurprisingly, the aforementioned examples of collective action are all within the field of reproductive health, a topic that is more traditionally associated with women’s health. Even urinary incontinence was framed as a complication of childbirth or a symptom of menopause. This is a reflection of one of the biases denounced by Inhorn and Whittle, namely the ‘reproductive essentialization of women’s lives’ [3]. One can indeed wonder if women’s voices are (more) heard or considered valid when they conform to this essentialist mould.

Issues that are not related to reproduction, such as misdiagnosing (or not diagnosing) women (white and of colour) with autism—the epidemiology and epistemology of which are still mostly based on white boys or young men—remain underexplored despite growing social media campaigns (#GirlsAreAutisticToo and #ActuallyAutistic). On different platforms, autistic women share their experiences of ‘masking’ which refer to minimising soothing behaviours that help them cope with sensory overstimulation in order to be viewed as not or less autistic [4]. Such insight is crucial to understand how autism manifests in women and therefore to arrive at a better epidemiology of autism. The call for intersectional and inclusive autism research would address another anti-feminist bias, and may find a clear response in social media collective action [5].

Collective action has also influenced how Covid-19 research is conducted in many regards. The pandemic has been another occasion to witness the power of women’s voices outside reproductive health, and the translation of their online activism in immediate policy changes and concrete research endeavour. For example, the acknowledgement of long-Covid as a disease (e.g. Survivor Corps, #LongCovid), which affects women disproportionately [6], is a prominent achievement.

## Mechanisms at play

From a theoretical and methodological perspective, we observe two powerful mechanisms at play on social media, which can facilitate the implementation of feminist epidemiological research: social media as a ‘third’ space and the power of groups.

Stadter has coined the term ‘e-third’ in analysing the relationship between the self, the other and the electronic object [7]. The ‘third’ in psychoanalysis refers to the intersubjective space created outside of the two subjectivities (the therapist and the client) which offers many opportunities for creativity, reverie and co-creation [8]. The e-third provides the capacity for connection, ‘copresence’, constancy of presence, and play [7]. Social media posts and open comments to surveys can be seen as inhabiting this third space, akin to what is said in-between doors, at the end of a therapy session. Just like in the psychoanalytic space, something that is off-the-record matters enormously and reveals much about a matter. Researchers somehow miss the opportunity to use the third spaces that people occupy.

Another existing space that researchers are seldom interested in are peer-groups. Due to the fact that shared lived experience is their organising principle, and consciousness raising is one of their goals, self-help peer-groups are the ideal terrain to generate bottom-up research priorities. The online groups and communities can be seen as an extension of these groups. To some extent, they provide a safe and emancipatory space, from where not-belonging individuals are excluded [9]. Online groups have the advantage of being more accessible, transnational, more visible and providing space for more inclusive memberships. We would argue that this could bring feminist epidemiology to scale.

## Research recommendations

Given the emancipatory power of social media, we propose recommendations and practical implications for leveraging the potential of online-sourced feminist epidemiology at different stages of the research process, and for increasing synergies between researchers and the community.

First, we recommend gathering insights from social media groups and hashtags. Groups and hashtags not only signify what really matters to people in terms of their health and how they form communities based on shared health experiences, they also make the gap between real-world concerns and research questions visible. Social media content is set to become an increasing part of digital epidemiology and can serve as a ground from which research questions can emerge. Respectful, invited and thorough analyses of online spaces would also serve as material for the comparison of researchers’ formulation of research questions with real-world priorities.

Second, we believe that social media feedback can be a precious contributor in a project’s pilot phase. Online spaces provide the perfect environment to invite feedback on data collection instruments and what those instruments mean to participants. Without asking for undue labour (or by giving due credit for the labour donated), a space could be carved for bottom-up shaping and refining of research instruments.

Third—and this approach is the most used so far, social media users can become themselves research participants. It is more and more common for researchers to recruit participants online, particularly for “hard-to-reach” or marginalised groups. However, there is still room to improve recruitment processes, so that they become really inclusive, more representative, do not objectify lived experience, and do not encroach on people’s safe spaces. Observing an ethics of the other that moves beyond procedural ethics and adopting research designs that facilitate continuous reflection and iterative consent (e.g. participatory research) allow for a non-othering approach [10]. This is particularly important in research with sexualised, ethnicised, or racialised groups whose subjectivities are underrepresented.

Fourth, we see potential in engaging with users on social media to disseminate research findings in a way that pays attention to patriarchal sociocultural contexts and power dynamics, mitigates risks for political recuperation (e.g. for research on abortion) and stigmatisation, and generates respectful discourse on studied populations.

## Arriving at feminist epidemiology

The proposed recommendations and their practical implications aim at bringing to scale a feminist approach to epidemiology and creating opportunities to co-design and co-produce research in an era which is increasingly shaped by online networks and spaces. By following this path, we may well witness the setting of new research priorities and the long-overdue advent of feminist epidemiology.

## Data Availability

Not applicable.
